# Laparoscopic Cholecystectomy in the Presence of Lumboperitoneal Shunt

**DOI:** 10.1155/2013/929082

**Published:** 2013-07-29

**Authors:** Alexandros Charalabopoulos, Abraham J. Botha

**Affiliations:** Department of General Surgery, Upper GI Unit, St Thomas' Hospital, Westminster Bridge Road, London SE1 7EH, UK

## Abstract

Laparoscopic cholecystectomy remains the mainstay of treatment in patients with gallstone disease. Nowadays more than ever before, patients present with more comorbidities and entities that make the laparoscopic approach composite. One of these is the presence of lumboperitoneal (LP) shunts. Herein, we describe a case of successful laparoscopic cholecystectomy in a patient with an LP shunt and an occipital nerve stimulator in the anterior abdominal wall. We describe alterations in technique, aiming at surgeons that perform laparoscopic cholecystectomies with useful tips in order to successfully deliver the operation. A brief review of the literature in the current subject is also given.

## 1. Introduction

A laparoscopic approach remains the technique of choice when performing a cholecystectomy. The reduction in postoperative pain, reduced hospital stay, and the cosmetic benefits have ensured its widespread use. With increasing use, surgeons are now being presented with patients in whom other medical comorbidities impact upon the feasibility of laparoscopic surgery.

Patients with hydrocephalus treated with a ventriculoperitoneal (VP) or lumboperitoneal (LP) shunt represent such a group. In these patients, a silicone catheter runs from the subarachnoid space in the ventricles of the brain (VP shunt) or the cauda equina of the spinal cord (LP shunt) and travels in a subcutaneous plane to eventually pass into the peritoneal cavity, into which they drain excess cerebrospinal fluid. In 1995, 33.000 patients required insertion of a VP shunt for hydrocephalus [1].

In this paper we report on a case of successfully performing a laparoscopic cholecystectomy with normal anaesthetic monitoring in the presence of an LP shunt. In addition to this we discuss the literature available on this topic.

## 2. Case Report

A 46-year-old lady was referred for investigation of a change in bowel habit. A computerised tomography (CT) colonography was performed and excluded any colonic pathology but did identify that the common bile duct was mildly dilated with a diameter of 8 mm, although no clear cause was found. Some reactive celiac lymph nodes were also seen and so the patient underwent an endoscopic ultrasound (EUS) scan to exclude a malignancy. The latter showed sludge and stones within the common bile duct and gallbladder. A subsequent endoscopic retrograde cholangiopancreatography-(ERCP-) guided sphincterotomy and balloon trawl of the common bile duct were performed leaving the common bile duct clear. Given the previous presence of gallstones within the common bile duct, it was decided to proceed to elective laparoscopic cholecystectomy. Laboratory investigations on the day before surgery revealed normal liver biochemistry (alkaline phosphatase 120 IU/L, bilirubin 3 umol/L, and alanine aminotransferase 23 IU/L).

**Figure 1:**
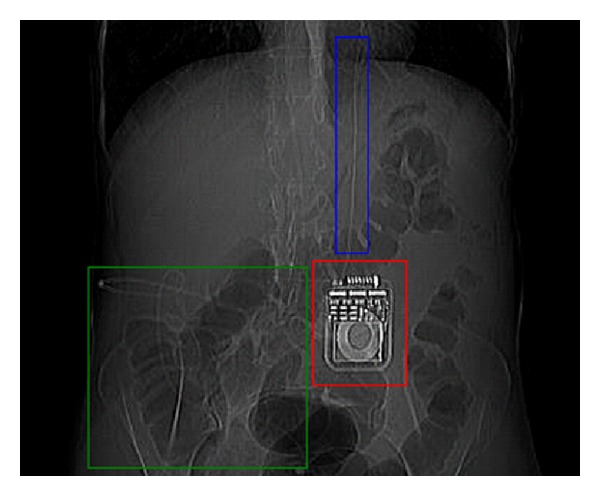
A preinsufflation “scout” view from the patient's CT colonography. Red box: the occipital nerve stimulator generator; blue box: wire from occipital nerve stimulator generator; and green box: course of lumboperitoneal shunt.

The patient's medical history was remarkable for excision of an intracranial epidermoid cyst in 1993. The patient developed nonobstructive hydrocephalus following this procedure and required the insertion of an LP shunt. The shunt ran from the subarachnoid space at the level of fourth and fifth lumbar vertebrae, around the right flank, through the right rectus abdominis muscle and peritoneum and the tip lay within the peritoneal cavity in the pelvis. In addition to the hydrocephalus, the patient suffered from chronic chemical meningitis and headaches, which were managed with an occipital nerve stimulator and oral analgesia. The generator for the occipital nerve stimulator lay within the subcutaneous tissue in the right paraumbilical region and the wires ran cranially in the subcutaneous plane from this generator (Figure 1). The patient otherwise had a history of well-controlled asthma, depression, gastroesophageal reflux disease, and coeliac disease.

The patient was admitted for an elective laparoscopic cholecystectomy under general anaesthesia. A number of modifications were made to the standard procedure for a laparoscopic cholecystectomy. Firstly, port placement was altered. A 10 mm port was created through the right rectus muscle 4 cm below and lateral to the umbilicus to avoid the occipital nerve stimulator generator. A Hasson's approach was used to place this port and this was then used to insufflate the peritoneal cavity. A further 10 mm port was placed in the midline within the epigastric region under direct vision. The light from the camera within the abdomen was used to identify the wire running cranially from the occipital nerve stimulator generator and thus ensure the epigastric port was placed safely. Two 5 mm ports were placed in the right side of the abdomen under direct vision to guide port placement around the LP shunt. These were sited within the right upper quadrant and the lateral aspect of the right abdomen at the level of the umbilicus. Secondly, the pneumoperitoneum pressure was set to 7 mmHg to minimise the risk of retrograde flow of gas along the LP shunt, carbon dioxide absorption into the blood, and change in venous pressure. This intervention was aimed at limiting any alteration in intracranial pressure during the procedure. Routine anaesthetic monitoring was used and no alterations were made to patient positioning with reverse Trendelenburg with the left tilt used during the procedure.

The patient tolerated the procedure well and there were no intraoperative complications. Postoperatively, the patient remained neurologically intact and there was no change in the frequency or severity of her headaches or other symptoms of raised intracranial pressure. The patient was discharged the day following the procedure. 

## 3. Discussion

We have described a case of successful laparoscopic cholecystectomy in the presence of an LP shunt with minimal modification in surgical technique. On review of the literature, there is only one reported case of successful laparoscopic cholecystectomy in the presence of an LP shunt [2].

Laparoscopic surgery in the presence of a VP shunt has, however, been widely reported and discussed. A retrospective review of urological laparoscopic surgery with standard anaesthetic monitoring in 18 patients with VP shunts revealed no untoward surgical, anaesthetic, or neurological events [3]. An average insufflation pressure of 16 mmHg was used. The authors did, however, identify the need for three VP shunts to be revised. A further case series of four patients reported no anaesthetic or neurological complications and no VP shunt revisions being required [4]. Insufflation pressures of 10–15 mmHg were used in this series. There are only two reported cases of unexpected complications from laparoscopic surgery in the presence of a VP shunt. The first was a case of shunt failure felt to be a result of impaction of soft tissue or air in the distal catheter, as a result of peritoneal insufflation [5]. The second reported was of a patient suffering ventilatory failure secondary to extensive subcutaneous emphysema after laparoscopic surgery in the presence of a recently placed VP shunt [6].

Cases of successful laparoscopic cholecystectomy with standard anaesthetic monitoring in the presence of a VP shunt have also been reported [7–9]. A retrospective case series of 23 patients with VP shunts undergoing laparoscopic cholecystectomy did, however, find that 35% of patients needed conversion to an open procedure owing to dense adhesions within the abdomen [10].

The proposed complications related to using laparoscopic surgery in the presence of a VP or LP shunt include the potential to increase intracranial pressure (ICP), cause shunt malfunction, and precipitate shunt infection. It has been proposed that the ICP rises during laparoscopic surgery due a ‘‘Valsalva-like” phenomenon resulting in cerebral vascular engorgement [11]. In this study on two children with Arnold-Chiari malformations (type II), Uzzo et al. saw a sudden increase of ICP by 12 mmHg to a maximum of 25 mmHg. This was matched by an increase in the flow rate of cerebrospinal fluid from the shunt and no adverse neurological effects were seen postoperatively. It has, however, been shown by Josephs et al. in a pigmodel, that there is an equivalent increase in ICP during laparoscopic surgery regardless of the baseline ICP, thus, questioning the clinical significance of Uzzo et al.'s findings [12]. Interestingly, the impact of patient positioning on ICP has been shown to be of similar importance to the presence/absence of a pneumoperitoneum [13]. Although increased arterial pCO2 secondary to absorption of carbon dioxide from the peritoneal cavity has been proposed as a possible mechanism for the rise in ICP, a number of studies have demonstrated a change in ICP secondary to a pneumoperitoneum in the presence of unchanged arterial pCO2 and pH readings [11, 12]. The potential for shunt malfunction and subsequent retrograde flow of gas along the shunt has been shown to be very unlikely. An in vitro model was used to test nine forms of VP shunt valves and demonstrated that none of the valves demonstrated any retrograde flow when exposed to pressures up to 350 mmHg [14]. Disruption of the seal on seven out of nine shunts was, however, seen at pressures above 80 mmHg. Ongoing flow of cerebrospinal fluid has been seen from in vivo VP shunts with a standard pneumoperitoneum pressure of 10–15 mmHg [4]. These findings suggest that previously suggested strategies of clamping or externalising the end of the VP shunt to minimise the risk of retrograde flow are likely to be unwarranted and could result in an increase in ICP due to blockage of normal cerebrospinal fluid flow [9]. Shunt infection remains a risk when performing laparoscopic surgery in the presence of a VP or LP shunt. A case series of 23 procedures by Allam et al. found two cases (9%) of postoperative shunt infection requiring shunt removal and replacement [10]. This series only included patients undergoing laparoscopic cholecystectomy and reported that a 9% infection rate was equivalent to that seen when other forms of laparoscopic surgery are performed in the presence of a VP shunt.

Although the aforementioned potential complications exist, there is now an increasing use of a laparoscopic approach to facilitate shunt placement and revision/replacement [15–17]. A key factor in support of this approach is the reduced postoperative morbidity and hospital stay associated with laparoscopic surgery.

In summary, performing a laparoscopic cholecystectomy in the presence of an LP shunt appears to be safe. Alterations in technique, such as reducing the pressure of the pneumoperitoneum and altering port placement, could help to reduce the risk of shunt-related complications. Given the potential risks of invasive ICP monitoring and the literature on laparoscopic surgery in the presence of VP shunts, direct monitoring of the ICP during laparoscopic surgery in the presence of LP shunts does not appear to be necessary. Further studies, however, are required to increase the limited evidence based on the safety of laparoscopic surgery specifically in the presence of LP shunts.
